# Effect of *Potato Virus Y* on the NADP-Malic Enzyme from *Nicotiana tabacum* L.: mRNA, Expressed Protein and Activity

**DOI:** 10.3390/ijms10083583

**Published:** 2009-08-13

**Authors:** Veronika Doubnerová, Karel Müller, Noemi Čeřovská, Helena Synková, Petra Spoustová, Helena Ryšlavá

**Affiliations:** 1 Department of Biochemistry, Faculty of Science, Charles University, Hlavova 2030, Prague 2, 128 40, Czech Republic; E-Mail: rysl@natur.cuni.cz (H.R.); 2 Institute of Experimental Botany, Academy of Sciences of the Czech Republic, Rozvojová 263, Prague 6, 165 02, Czech Republic; E-Mail: muller@ueb.cas.cz (K.M.); 3 Institute of Experimental Botany, Academy of Sciences of the Czech Republic, Na Karlovce 1a, Prague 6, 160 00, Czech Republic; E-Mails: cerovska@ueb.cas.cz (N.C.); synkova@ueb.cas.cz (H.S.); spoustova@ueb.cas.cz (P.S.)

**Keywords:** NADP-malic enzyme, *Nicotiana tabacum* L., *Potato virus* Y strain NTN (PVY^NTN^), *Potato virus* Y strain O (PVY^O^), biotic stress, real time-PCR

## Abstract

The effect of biotic stress induced by viral infection (*Potato virus Y*, strain NTN and O) on NADP-malic enzyme (EC 1.1.1.40) in tobacco plants (*Nicotiana tabacum* L., cv. Petit Havana, SR1) was tested at the transcriptional, translational and activity level. The increase of enzyme activity in infected leaves was correlated with the increased amount of expressed protein and with mRNA of cytosolic NADP-ME isoform. Transcription of the chloroplastic enzyme was not influenced by viral infection. The increase of the enzyme activity was also detected in stems and roots of infected plants. The effect of viral infection induced by *Potato virus Y*, NTN strain, causing more severe symptoms, was compared with the effect induced by milder strain PVY^O^. The observed increase in NADP-malic enzyme activity in all parts of the studied plants was higher in the case of PVY^NTN^ strain than in the case of strain PVY^O^. The relevance of NADP-malic enzyme in plants under stress conditions was discussed.

## 1. Introduction

Viral diseases are one of the main causes for decreased world-wide crop productivity [1]. On the other hand plants have both preformed and infection-induced factors, that help them to defend against pathogen infestation. Whereas preformed factors are provided by waxy cuticular “skin” layers and anti-microbial compounds, induced defence responses are connected partly with specific responses (*e.g.,* generation of reactive oxygen species, synthesis of NO, opening of ion channels, modification of protein phosphorylation status, hypersensitive cell death and transcriptional activation of numerous defence-related genes), but also with changes of fluxes through primary and secondary metabolic pathways [2,3]. Under stress conditions enzymes with anaplerotic function could play more important role.

In the present study, the influence of biotic stress caused by plant viruses on non-photosynthetic NADP-malic enzyme (E.C. 1.1.1.40) (NADP-ME) from C_3_ plant *Nicotiana tabacum* L. was investigated. A relationship between the non-photosynthetic isoform of NADP-ME and plant defence response was suggested in our previous work and also by other authors [4–10]. NADP-ME catalyzes the oxidative decarboxylation of l-malate using NADP^+^ as a coenzyme in the presence of divalent metal ions (Mg^2+^ or Mn^2+^ ions) to produce pyruvate, NADPH and CO_2_ [11,12]. The presence of a cofactor and the coenzyme is required for the catalysis. The NADP-ME is widely distributed in nature from bacteria to humans, in both the cytosol and the mitochondria [13]. Animal cytosolic NADP-ME is involved in the generation of NADPH necessary for the biosynthesis of fatty acids and steroids in liver and adipose tissues. This isoform may also participate in microsomal drug detoxification [14]. Besides this, NADP-ME in plants is also localized in chloroplasts, well known is its photosynthetic function in some C_4_ plants (*e.g.,* maize). Supporting functions such as providing NADPH for assimilatory process (*e.g.,* lipid biosynthesis), maintaining intracellular pH [11] and above-mentioned implication in plant defence to stress are proposed for non-photosynthetic C_3_ NADP-ME isoform localized in chloroplasts and/or in cytosol [12,15]. NADPH production is also necessary for biosynthesis of specific defence compounds (*e.g.,* phytoalexins, lignins, osmolytically active compounds) important under stress [12] or act as cofactor for antioxidative enzymes [3].

The structure of plant and animal NADP-MEs is predominantly tetrameric, with relative molecular mass of one subunit ranging from 62,000 to 67,000 [12,15,16]. NADP-malic enzymes in plants are encoded by a small gene family. Family of NADP-MEs from plants, in which the whole genome sequence is known (C_3_ model plants *Arabidopsis thaliana* [17] and rice [8]), is in both composed of three genes encoding cytosolic and one gene encoding plastidic isoform of NADP-ME. Two recombinant isoforms of *Nicotiana tabacum* L. were fully characterized (chloroplastic Nt-NADP-ME1 with predicted mature molecular mass 63,300 and cytosolic Nt-NADP-ME2 with relative molecular mass 65,400) and the transcript of the third putative NADP-ME has been identified [15].

In plants, the photosynthetic isoform (NADP-ME from maize leaves) is up regulated by light [18] and inhibited by an excess of l-malate as a substrate or by other compounds (the strongest inhibition was observed in the presence of oxaloacetate and α-ketoglutarate) [11]. Recombinant non-photosynthetic isoforms of NADP-ME from *Arabidopsis thaliana* and *Nicotiana tabacum* L. were found to be regulated by some citric acid cycle intermediates (oxaloacetate, fumarate, succinate) and ATP [15,19].

This study is focused on changes in transcriptional, expressional and activity levels of non-photosynthetic tobacco NADP-ME under biotic stress caused *by Potato virus Y*, (PVY), one of the most economically damaging plant viruses [20].

We proceeded in our previous work, which proved five times enhanced activity of NADP-ME in tobacco leaves during infection caused by *Potato virus Y –* NTN strain, whereas the effect of *Potato virus A* on NADP-ME activity was only mild [6]. The aim of this study was found out: i) correlation between severity of PVY^NTN^ and PVY^O^ symptoms and NADP-ME activity; ii) influence of viral infection on NADP-ME activity in particular plant organs (leaf, stem, roots); iii) relationship between the enhanced activity of NADP-ME and its expression; iv) evaluation of the role of cytoplasmic and chloroplastic isoforms of NADP-ME for the enhanced accumulation of NADP-ME.

## 2. Results and Discussion

### 2.1. Course of Viral Infection Caused by PVY^O^ and by PVY^NTN^ in Nicotiana tabacum L. Plants

Generally, the study of biotic stress is more complicated than abiotic stress, due to problems with the quantification of the stressor. To test the impact of different severity of viral disease on metabolic changes in tobacco plants, we used two strains of PVY characterized with different severity of symptoms. *Potato virus Y*, strain NTN (PVY^NTN^) is harmful, economically significant, rapidly expansive virus, which caused extensive veinal necrosis of leaves of host tobacco plants. *Potato virus Y*, strain O (PVY^O^) caused milder symptoms (mottle mosaic, chlorosis) than PVY^NTN^. Furthermore, the both used isolates of PVY had the capacity to infect tobacco systemically [21].

**Figure 1.**
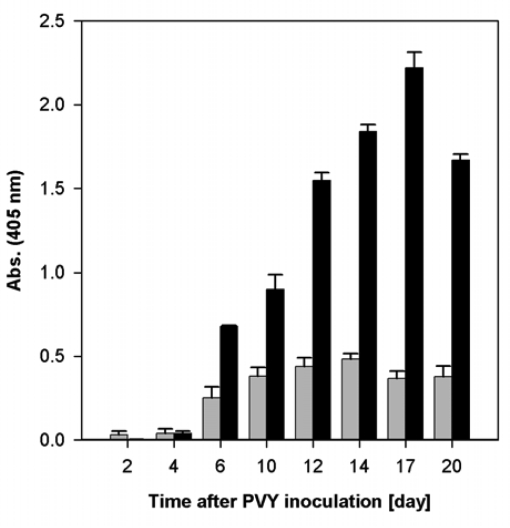
The relative content of PVY^O^ (grey columns) and PVY^NTN^ (black columns) in leaves of *Nicotiana tabacum* L. in the course of infection. The relative virus content was determined by DAS-ELISA with *p*-nitrophenylphosphate as substrate for alkaline phosphatase. Absorbance at 405 nm is proportional to the virus content. The absorbance of each sample was measured in triplicate, S.D. are shown.

Determinations of relative content of virus in tobacco leaves by DAS-ELISA in the course of viral infection confirmed its increase. In the case of more harmful virus (PVY^NTN^), higher relative content of virus was determined than in the case of PVY^O^ (Figure 1). The maximal content of both viruses was found ca 12^th^–17^th^ day after inoculation (Figure 1).

### 2.2. Influence of PVY Infection on NADP-ME Activity

**Figure 2.**
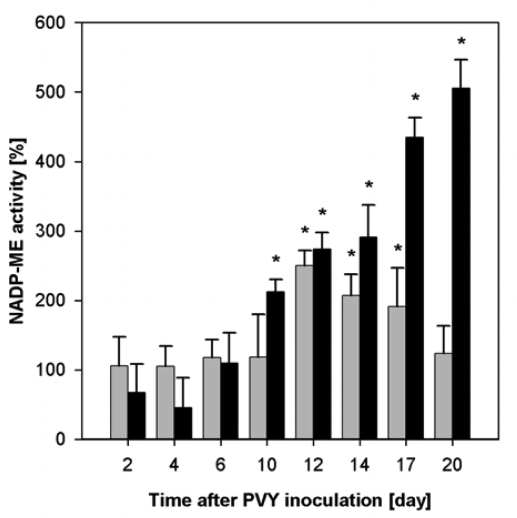
Activities of NADP-ME from *Nicotiana tabacum* L. leaves during PVY^O^ (grey columns) and PVY^NTN^ (black columns) infections calculated per fresh mass. The activity of each sample is shown as percentage of the non-infected control in particular day, where 100% NADP-ME is 0.053 ± 0.017 μmol·min^−1^·g^−1^. NADP-ME activity in mock-inoculated leaves (not shown) was the same as in non-inoculated controls. The activity was measured in at least three samples, S.D. are shown. Statistical analysis was done using t-test. * denotes significant difference from controls at P<0.05.

Enhanced NADP-ME activity was found in infected tobacco plants. The NADP-ME activity in PVY^O^ infected leaves was 2.5-fold higher than in control plants on the 12^th^ day after the inoculation. Similarly, a 5-fold enhanced NADP-ME activity in PVY^NTN^ infected leaves was detected on the 20^th^ day (Figure 2). In the course of infection, high virus content correlated with enhanced activity of NADP-ME (Figures 1, 2). Mock-inoculation did not change NADP-ME activity in leaves; it was the same as in leaves of healthy control plants (data not shown).

**Figure 3.**
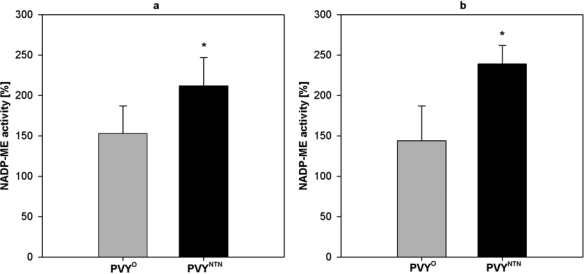
Activities of NADP-ME from *Nicotiana tabacum* L. stems **(a)** and roots **(b)** collected 11^th^ day after PVY^O^ inoculation and 13^th^ day after PVY^NTN^ inoculation calculated per fresh mass. The activity of each sample is shown as percentage the activity value of the non-infected control, where 100% NADP-ME is in stems 0.040 ± 0.005 μmol·min^−1^·g^−1^, in roots 0.076 ± 0.016 μmol·min^−1^·g^−1^. The activity was measured in at least six samples, S.D. are shown. Statistical analysis was done using t-test. * denotes significant difference from controls at P<0.05.

NADP-ME activity in stems and roots (Figure 3) was measured in a period, when symptom development reached its maximum (*i.e.,* 11^th^ day of PVY^O^ and 13^th^ day of PVY^NTN^ infection). NADP-ME activity in stems was 1.5-fold higher in plants infected by milder PVY^O^ and 2.1-fold higher in plants infected by PVY^NTN^ compared with control stems (Figure 3a). Similar situation was observed in roots: PVY^O^ caused 1.4-fold increase in NADP-ME activity, whereas PVY^NTN^ caused 2.4-fold increase in NADP-ME activity as compared with control roots (Figure 3b).

In our previous study, when we tested the influence of *Potato virus* A (PVA) that caused only very mild symptoms, moderate increase of NADP-ME activity was found in upper (systemically infected) leaves of *Nicotiana tabacum* L. [6]. In these leaves the highest PVY content was found. This finding was in correlation with the highest increase of NADP-ME activity (Figure 2). Lower increase of NADP-ME activity was found in roots, where the virus content was lower than in leaves (Figure 3; data not shown). We can conclude that activity of NADP-ME increased in correlation with development of potyviral infection in tobacco plants, nevertheless, it was dependant on the type of *Potyvirus* used for inoculation (Figures 1,2; [6]).

**Figure 4.**
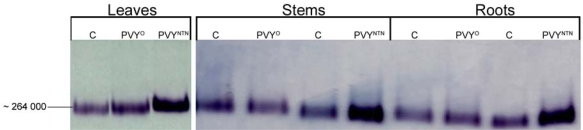
Detection of NADP-ME in 10% polyacrylamide gel after non-denaturating electrophoresis. Control (marked as C), PVY^O^ and PVY^NTN^ infected leaves, stems and roots of *Nicotiana tabacum* L. plants were analysed. Samples were collected at the maximal symptoms occurrence (11^th^ day after PVY^O^ inoculation and 13^th^ day after PVY^NTN^ inoculation).

NADP-ME activity was also evaluated after electrophoretic separation in polyacrylamide gel under native conditions. Higher NADP-ME activity in infected plants corresponded to more intensive bands noticeable after its detection (Figure 4). Active NADP-ME zones in gel regarding to control, PVY^O^ and PVY^NTN^ infected leaves; stems and roots are shown in Figure 4. One band was visible in all parts (leaves, stems and roots) of *Nicotiana tabacum* L. (Figure 4). The estimation of relative molecular mass after native electrophoresis was performed according to Ferguson’s method (using a set of various polyacrylamide gel concentrations) and was approximately 264,000. This molecular weight would correspond to tetramer and to the cytosolic NADP-ME. Particularly isoforms could not be distinguished, probably due to that they have similar molecular weights or due to low activity of chloroplastic isoform (data not shown). No band of NADP-ME activity was observed in control gel incubations without substrate, coenzyme and cofactor, respectively (results not shown). Different results were found in *Nicotiana benthamiana* plants, where PVY^NTN^ did not cause a significant increase of the NADP-ME activity in leaves, but only a slight increase (1.8-fold higher) of NADP-ME activity in roots [22]. *Nicotiana benthamiana* and *Nicotiana tabacum* L. plants also differed in NADP-ME isoform content. Generally, we can conclude, that enhanced activity of NADP-ME was probably not a general response to potyviral infection in all host plants, seeing that the presence of isoforms and their functions might be also important.

However, correlations between NADP-ME activity and abiotic stress conditions were also observed in other systems. Increased activity of NADP-ME was found in *Phaseolus vulgaris* plants after Cd application [10], in hyperhydric carnation shoots cultured for 14 and 28 days *in vitro* [23], in leaves of submersed aquatic species *Egeria densa* after transfer from low temperature and light to high temperature and light conditions [24], in cold hardened winter rye (*Secale cereale* L.) [25], in leaves of olive plants under salt stress condition [26], in leaves of *Aloe vera* L. under salt stress [7], similarly, salt and osmotic stress induced NADP-ME in leaves and roots of rice seedlings [8,27].

Müller *et al*. in addition to wide scale of abiotic stressors (hypoxia, NaCl, Na_2_CO_3_, NaHCO_3_, abscisic acid, polyethylenglycol, UV-B exposure), also studied the influence of several isolated biotic stressors (fungal elicitors, cellulase and infiltration with *Agrobacterium tumefaciens*) on NADP-ME levels in *Nicotiana tabacum* L., but it was not done during the course of any real infection [15]. Various stressors affect NADP-ME specific activity in leaves, stems and roots in different manners. The most significant increase of specific activity in tobacco leaves was found after polyethylene glycol, NaHCO_3_, UV-B exposure and hypoxia treatment. Enhanced specific activity of NADP-ME was also found in stems after cellulase treatment and in leaves after *Agrobacterium tumefaciens* infiltration, but in roots specific NADP-ME activity was rather lower than in control plants [15]. Contrary, increased NADP-ME activity was found in roots of maize seedlings treated with cellulase, jasmonate and fungal elicitor [4].

### 2.3. Influence of PVY^NTN^ Infection on the Expression of NADP-ME

**Figure 5.**
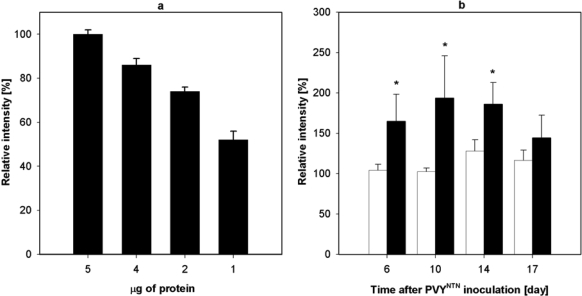
Immunochemical detection of NADP-ME. (a) Test of linearity of Western blot analysis signal using various quantities of purified NADP-ME protein from *Nicotiana tabacum* L. leaves. Estimated relative molecular mass according to standard protein was 66,000. Plot indicates decrease in relative intensity of signal (expressed in %) evaluated densitomerically by Elfoman 2.0. (b) Detection of NADP-ME protein in control (white columns) and PVY^NTN^ infected (black columns) leaves collected 6^th^–17^th^ day after inoculation by Western blot analysis. At least four Western blot analyses were performed. 100% corresponds to intensity of protein NADP-ME in control sample in the 6^th^ day. Statistical analysis was done using t-test. * denotes significant difference from controls at P<0.05.

The amount of NADP-ME protein was tested immunochemically. Western blot analysis was used for the detection of NADP-ME in tobacco leaves the 6^th^–17^th^ day after PVY^NTN^ inoculation. For this purpose, rabbit antibodies raised against NADP-ME from maize seeds were used. The linearity of the response was tested using various amounts of NADP-ME purified from *Nicotiana tabacum* L. leaves using three types of chromatography (DEAE-cellulose, Sephacryl S 300 and 2′,5′-ADP-Sepharose 4B) according to Ref. [28] and Figure 5a.

More intensive bands that proved enhanced expression of NADP-ME were found from the 6^th^ till the 17^th^ day of viral infection (Figure 5b). Intensity of bands was analyzed densitometrically using commercial program Elfoman 2.0. In comparison with control plants approximately 2-fold higher signal was found in PVY^NTN^ infected leaves. The cytosolic and chloroplastic isoforms cannot be distinguished immunochemically because the antibodies are not specific to NADP-ME isoforms. Our former experiments confirmed that the contribution of chloroplastic isoform to total enzyme activity is very low. From this finding we deduce that the amount of corresponding protein is also inconsiderable (data not shown).

### 2.4. Influence of PVY^NTN^ Infection on Transcription of NADP-ME

**Figure 6.**
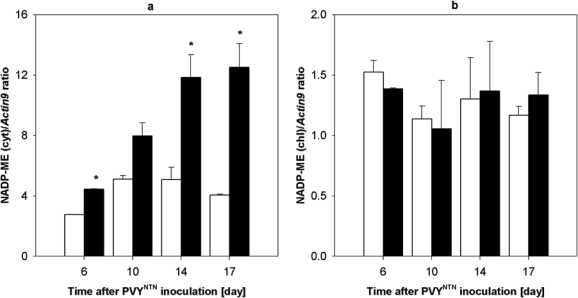
Quantification of cytosolic and choroplastic NADP-ME mRNA. (a) Amount of cytosolic and (b) chloroplast NADP-ME mRNA in PVY^NTN^ infected *Nicotiana tabacum* L. leaves (black columns) compared to healthy control (white columns) leaves measured by reverse transcription followed by real-time PCR method 6^th^ – 17^th^ day of viral infection. Result corresponds to ratio of cytosol NADP-ME transcript (a) or chloroplast NADP-ME transcript (b) and standard gene *Actin9*. The amount of NADP-ME mRNA was calculated from at least three samples, S.D. are shown. Statistical analysis was done using t-test. * denotes significant difference from controls at P<0.05.

To find out a relationship between the enhanced activity of NADP-ME in *Nicotiana tabacum* L. plants caused by potyviral infection and the enzyme biosynthesis *de novo*, we evaluated also the amount of NADP-ME mRNA (Figures 5 and 6). Reverse transcription followed by real-time PCR was used to measure transcription of NADP-ME mRNA in tobacco leaves in the period from 6^th^ to 17^th^ day after PVY^NTN^ inoculation. *Actin9* was used as standard gene as its transcription was not affected by biotic stress [29]. In infected samples the increase of amount of NADP-ME mRNA of cytosolic isoform was observed (Figure 6a). Maximum transcription occurred the 17^th^ day after virus inoculation and was 3-fold higher compared to healthy control (Figure 6a). However, the transcription of chloroplastic isoform of NADP-ME was not influenced by PVY^NTN^ infection (Figure 6b).

This means that tobacco leaves cytosolic NADP-ME isoform should be involved in plant defence response against potyviral infection, whereas chloroplastic one probably not. Melting curve analysis of real-time PCR reaction showed a single product peak in the expected temperature range (result not shown). Products of real time PCR using primers for chloroplastic and cytosolic NADP-ME were sequenced. PCR fragments showed 100% homology with GenBank sequences DQ923119 and DQ923118, respectively.

The biosynthesis of the enzyme may not be the only source of the enhanced activity of NADP-ME in infected tobacco leaves, but other factors can modulate the activity, too, *e.g.,* the interaction with heat shock protein 70 [30] that is associated with some types of stress [31]or some other factors.

NADP-ME transcription level after exposure to stress condition was also studied by other authors. Relative transcription of chloroplastic NADP-ME isoform in *Nicotiana tabacum* L. leaves dramatically increased after fungal elicitor and after *A. tumefaciens* infiltration treatment [15]. On the contrary, treatment by cellulase caused decrease in transcription of chloroplastic NADP-ME mRNA in leaves, stems and roots. Relative transcription of cytosolic NADP-ME mRNA was higher in roots after cellulase treatment, higher in leaves after fungal elicitor treatment and in leaves after A. *tumefaciens* infiltration [15]. Abiotic stressors affected in different manner particular *nadp-me* transcripts [15]. In leaves of rice plants grown in carbonates (NaHCO_3_ and Na_2_CO_3_) the increased activity by more than 50% was accompanied by gene NADP-ME_2_ induction [27]. Using transgenic plant *Arabidopsis thaliana* with inserted gene for rice cytosolic NADP-ME_2_, the role of this gene in adapting to carbonate stress was confirmed [27]. The four rice NADP-ME genes all responded to salt and osmotic stresses regardless of subcellular protein localization [8]. Enhanced activity in roots of maize seedlings treated with cellulase, jasmonate and fungal elicitor corresponds to increased NADP-ME protein and mRNA [4].

### 2.5. Proposed Function of NADP-ME during Viral Infection in Plants

The reason for the significantly enhanced activity of NADP-ME in tobacco plants during viral infection compared to control plants is probably the production of NADPH. The supply of reducing equivalents in the form of NADPH is required in plant metabolism especially under stress conditions for biosynthesis of defence compounds such as flavonoids, phytoalexins and lignins [12,32]. Biotic stress is associated with the production of reactive oxygen species, which are formed in the reaction catalyzed by NADPH oxidase, with NADPH as coenzyme [3]. NADPH moreover could participate in the ascorbate – glutathione cycle for protection against oxidative damage [3].

**Figure 7.**
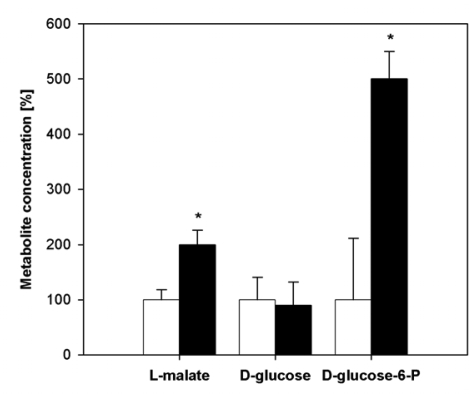
Concentrations of important cell metabolites. Measurement of concentrations of L-malate, D-glucose and D-glucose-6-phosphate in *Nicotiana tabacum* L. leaves of healthy plants and those infected by PVY^NTN^. Relative concentrations (expressed as % of healthy controls) are presented (L-malate: 100% corresponds to 10.5 mmol·g^−1^ F.W.; D-glucose: 100% corresponds to 2.8 mmol·g^−1^ F.W.; D-glucose-6-phosphate: 100% means 0.06 mmol·g^−1^ F.W.). Statistical analysis was done using t-test. * denotes significant difference from controls at P<0.05.

Enhanced NADP-ME activity corresponded also to higher concentration of l-malate in infected plants (Figure 7). Another source of NADPH is the reaction catalyzed by d-glucose-6-phosphate dehydrogenase. Concentration of d-glucose-6-phosphate was enhanced in the maximum of PVY^NTN^ symptom development (Figure 7). Whereas substrates of NADP-dependent enzymes (l-malate and d-glucose-6-phosphate) were approximately 2- and 4-fold, respectively higher in PVY^NTN^ infected tobacco leaves (Figure 7); concentration of d-glucose was similar in infected and healthy plants.

In tobacco plant under conditions of viral infection, stomatal conductance is reduced, the stomata are closed [6] and the CO_2_ supply for photosynthesis is limited. Under such conditions, CO_2_ formed in the reaction catalyzed by NADP-ME could be beneficial and it could help to balance substrate deficiencies. This hypothesis is supported by the fact that enhanced activities of phosphoenolpytuvate carboxylase and pyruvate, phosphate dikinase besides NADP-ME were found in stressed tobacco plants [6,33]. These enzymes together with NAD-malate dehydrogenase could hypothetically form a cycle, where NADH and energy in the form of ATP are consumed in this cycle, but at the same time NADPH is produced. These reactions are analogous to plants with “C_4_” or “single cell C_4_” photosynthesis [34], although besides CO_2_ accepted from the air, metabolic CO_2_ could be used. CO_2_ could be also released from l-malate transported from non-photosynthetic plant parts especially from roots, where also enhanced NADP-ME activity was found (Figure 3), as suggested by Hibberd and Quick [35]. In addition, production of CO_2_ by NADP-ME means the change of the ratio CO_2_ and O_2_, thus the enhanced photorespiration in infected cells could be relatively limited.

## 3. Experimental Section

### 3.1. Plant Material

*Nicotiana tabacum* L., cv. Petit Havana SR1 plants were grown in a greenhouse under 22/18 °C day/night temperatures. Day irradiance [overall integrated mid-values were *ca.* 500 μmol (quantum) m^−2^ s^−1^] was prolonged by the additional irradiation [PPFD *ca*. 200 μmol (quantum) m^−2^ s^−1^, Philips TL-D 36W/54-76 T] to 16 h. Seeds were sown in pots with sand and plantlets were transferred to soil after three weeks. Leaves of seven-weeks old plants were mechanically inoculated with *Potato virus Y,* strain *NTN* (PVY^NTN^) or *Potato virus Y*, strain O (PVY^O^) kindly provided by Dr. Dědič (Potato Research Institute, Havlíčkův Brod, Czech Republic).

Two groups of control plants were used for measuring of NADP-ME activities. The first one involved 50 healthy, non-inoculated plants, the second one included 50 mock-inoculated (buffer and carborundum) plants. The samples were collected from control and infected leaves, stems and roots within three weeks. Leaves were collected each 2–4 days during infection until plants death, whereas stems and roots were collected in the maximum of symptom development (11^th^ and 13^th^ day, respectively). Roots were washed thoroughly and dried. Material from several plants was cut with scissors, mixed and packet separately as 0.5–2 g sample. Healthy and mock-inoculated plants were used as controls. Samples were immediately frozen in liquid N_2_ and stored at −80 °C. The extent of viral infection was determined by DAS-ELISA [36] in homogenates of the leaves of infected plants using polyclonal antibodies raised against the respective pathogens [20]. Two different biological experiments with PVY^O^ and three independent experiments with PVY^NTN^ were performed.

### 3.2. Enzyme Activity Assay

For assay of NADP-ME activity, 0.5 g of plant tissue were homogenized in 1.5 mL of 100 mM Tris-HCl buffer (pH 7.8) containing 1 mM dithiothreitol, 1 mM EDTA and 5 mM MgCl_2_ (buffer A); then 0.02 g of polyvinylpolypyrrolidone was added and the homogenate was centrifuged at 23,000× *g* for 15 min at 4 °C. The supernatant was used as plant extract for measuring NADP-ME activity. The NADP-ME activity was monitored spectrophotometrically as a change of absorbance at 340 nm at 25 °C and activities were calculated as μmol of substrate converted per minute and per gram of fresh weight. The NADP-ME assay mixture contained 100 mM Tris-HCl buffer (pH 7.4), 10 mM l-malate, 2 mM MgCl_2_ and 0.2 mM NADP^+^ in total volume of 1 mL. The reaction was started by addition of 50 μL of the enzyme extract [6].

### 3.3. Native PAGE

Native gel electrophoresis was performed according to Lee and Lee [37]. The same volume (25 μL) of all plant extracts in 20% (w/v) of sucrose was applied to the gel. Protein zones with NADP-ME activity were detected after incubation of polyacrylamide gels (10%) in a solution of 100 mM Tris-HCl (pH 7.4) containing 10 mM l-malate, 10 mM MgCl_2_, 2 mM NADP+, 0.1 mg/mL nitroblue tetrazolium and 5 μg/mL phenazine methosulfate at room temperature [4]. The estimation of relative molecular mass after native electrophoresis was performed according to Ferguson’s method [22,38].

### 3.4. Purification of NADP-ME from Maize Seeds

NADP-ME from maize seeds was purified as was described previously for NADP-ME from tobacco leaves with some minor modification [28]. One hundred g of dry maize seeds were finely ground and then extracted in 300 mL of buffer A (100 mM Tris-HCl, pH 7.8 containing 1 mM dithiothreitol, 1 mM EDTA and 5 mM MgCl_2_) for 30 min. Subsequent procedures including ion-exchange, gel and affinity chromatography were performed by the same way as in Ref. [28]; only Sephadex G 200 was replaced by Sephacryl S 300. The obtained enzyme preparation was used for rabbit immunisation. Similarly, purified NADP-ME from *Nicotiana tabacum* L. leaves [28] was used as a positive control and for quantification.

### 3.5. Preparation of Rabbit Antibodies against NADP-ME

Antibodies against NADP-ME were obtained by immunization of two New Zealand rabbits with 0.2 mg of the purified protein NADP-ME from maize seeds emulsified in Freund’s complete adjuvant, in subcutaneous and intramuscular injections. Booster injections (0.4 and 0.6 mg) of the same protein with incomplete adjuvant were applied after 21 and 42 days. Pure antigen (0.822 mg) was administered after 63 and 84 days. The rabbits were bled 14 days after the last antigen injection. The serum fractions were collected and stored at −20 °C until required. The immunoglobulin (IgG) fraction from the antisera was obtained according to [39] and stored in 0.05% (w/v) NaN_3_ at 4 °C.

### 3.6. Western Blot Analysis

Equal amounts of total proteins were subjected to SDS-polyacrylamide gel electrophoresis (PAGE) [40] in 10% polyacrylamide gels. After electrophoresis proteins were transferred to nitrocellulose membrane by electroblotting using a semi-dry system (Fastblot B31, Biometra). The immunochemical detection of NADP-ME was carried out using polyclonal rabbit antiserum raised against maize seed NADP-ME. Bound antibodies were visualized using mouse antirabbit IgG labelled with alkaline phosphatase. NBT-BCIP (nitroblue tetrazolium, 5-bromo-4-chloro-3-indolyl phosphate) was used as a substrate for alkaline phosphatase. At least four Western blot analyses were performed with control and infection samples. The linearity of band intensity was tested using various quantities of NADP-ME protein purified from tobacco leaves and determined by program Elfoman 2.0.

### 3.7. mRNA Extraction and Quantification

Total mRNA was isolated from 100 mg of leaf sample using RNeasy kit (Qiagen). Any contaminated genomic DNA was removed by treatment with DNaseI (Qiagen). The quantity and purity of the mRNA was determined spectrophotometrically and the level of intact RNA was determined by agarose gel electrophoresis.

Approximately 1 μg of RNA was transcribed by M-MLV reverse transcriptase (Promega) using oligo-dT primer. Quantitative real-time PCR was performed on a Lightcycler 480 (Roche) using FastStart DNA Master SYBR Green PLUS kit (Roche Applied Science). Conditions for the amplification of *Actin9*, NADP-ME(cyt) and NADP-ME(chl) transcripts were: 10 min polymerase activation at 95 °C and 50 cycles, each cycle at 95 °C for 10 s, 60 °C for 10 s and 72 °C for 15 s. Primers for chloroplastic and cytosolic form of NADP-ME were designed according to GenBank sequences DQ923119 and DQ923118, respectively. Sequences of primers were:

**Table:** 

MEcyt Forward	ATA CAT TCT TGT TCC TCG GAG CAG
MEcyt Reverse	CCC TTT GAA TCC ACC AGC CA
MEchl Forward	GCT CTC TTT ATA CAC TGC TCT GG
MEchl Reverse	AAG TTC GGC ATA TTC CTG TCC T

*Actin9* primers were kindly provided by Dr. Helena Štorchová (Institute of Experimental Botany, Academy of Sciences, Czech Republic). The ratio corresponding to the difference in NADP-ME transcription between healthy and infected plants was calculated according to Equation 1:

(1) $$\text{ratio}=\frac{e_{NADP-ME}^{CP_{NADP-ME}}}{e_{\text{Actin}9}^{CP_{\text{Actin}9}}}$$

where *e_NADP-ME_* and *e_Actin9_* represent PCR amplification efficiency of each product and *CP* represent the crossing point of each sample reaction.

### 3.8. Metabolite Concentration Measurements

Tobacco leaf samples were homogenized in 1 M HClO_4_ and centrifuged at 20,000g for 15 min. The supernatant was neutralized using 5 M K_2_CO_3_ to approx. pH 7. Precipitated KClO_4_ was removed by centrifugation. Metabolite contents were measured enzymatically based on methods described by Stitt *et al.* [41]. For the determination of d-glucose and d-glucose-6-phosphate, the reaction mixture contained 100 mM Tris–HCl (pH 8.1), 5 mM MgCl_2_, 1 mM ATP and 0.8 mM NADP^+^ in a total volume of 1 mL. The reaction was started adding 0.1 U of d-glucose-6-phosphate dehydrogenase and 0.1 U hexokinase. Increasing absorbance at 340 nm was monitored spectrophotometrically. d-Glucose-6-phoshpate was determined through the same reaction lacking the hexokinase and ATP. For the determination of malate, the reaction mixture contained 100 mM Tris-HCl (pH 8.1), 0.2 mM NAD^+^, 0.05 U of malate dehydrogenase, 5 U l-aspartate transaminase and 2 mM l-aspartate. Increasing absorbance at 340 nm was monitored spectrophotometrically. Concentrations of metabolites in samples were calculated from calibration curves using known concentrations of corresponding metabolite.

### 3.9. Statistical Analysis

Two independent PVY^O^ and three PVY^NTN^ experiments were performed. Leaves for enzyme activity measurements and real-time PCR quantification were processed at least in three samples in each experiment, activity of NADP-ME in roots and stems at least in six samples. Statistically significant differences in the mean values were tested by Student’s t-test at P=0.05.

## 4. Conclusions

Our results obtained in experiments dealing with real biotic stress caused by two strains of PVY documented in detail NADP-ME activity in various parts of plants in the course of infection. We found out that two strains of PVY, which differed in the severity of symptoms, both increase NADP-ME activity in leaves, stems and roots; milder strain PVY^O^ less than necrotic PVY^NTN^.

The PVY^NTN^ infection stimulated *de novo* synthesis of cytosolic NADP-malic enzyme in *Nicotiana tabacum* L. leaves, whereas chloroplastic isoform was not affected.

Many plants response to various types of stress by changes in the activity of NADP-ME, but its enhancement and protein expression vary among plant species and probably could correspond to plant tolerance to stress, however many experiments in this field are needed.
